# Supplementary material for: Muscle Proteomic Profile before and after Enzyme Replacement Therapy in Late-Onset Pompe Disease
Source: Int J Mol Sci. 2021 Mar 11;22(6):2850. doi: 10.3390/ijms22062850 (PMC8001152; doi:10.3390/ijms22062850)

Figure S2. Western blot full images

Fig. 2B

GLUL (45 kDa)
Santa Cruz 6640
gel 8%

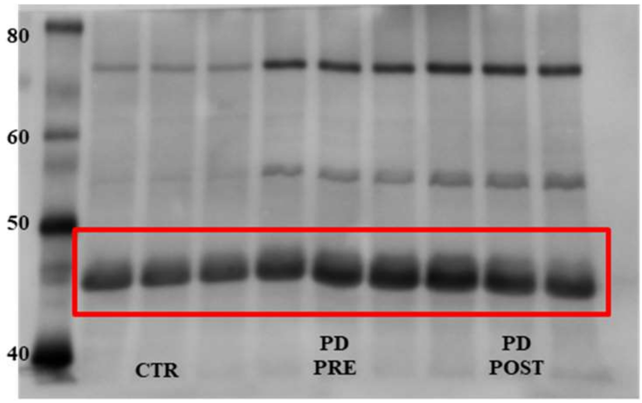

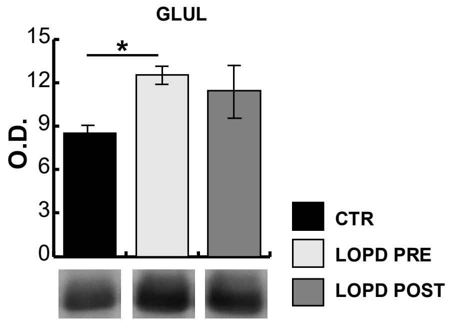

Total stain 8%

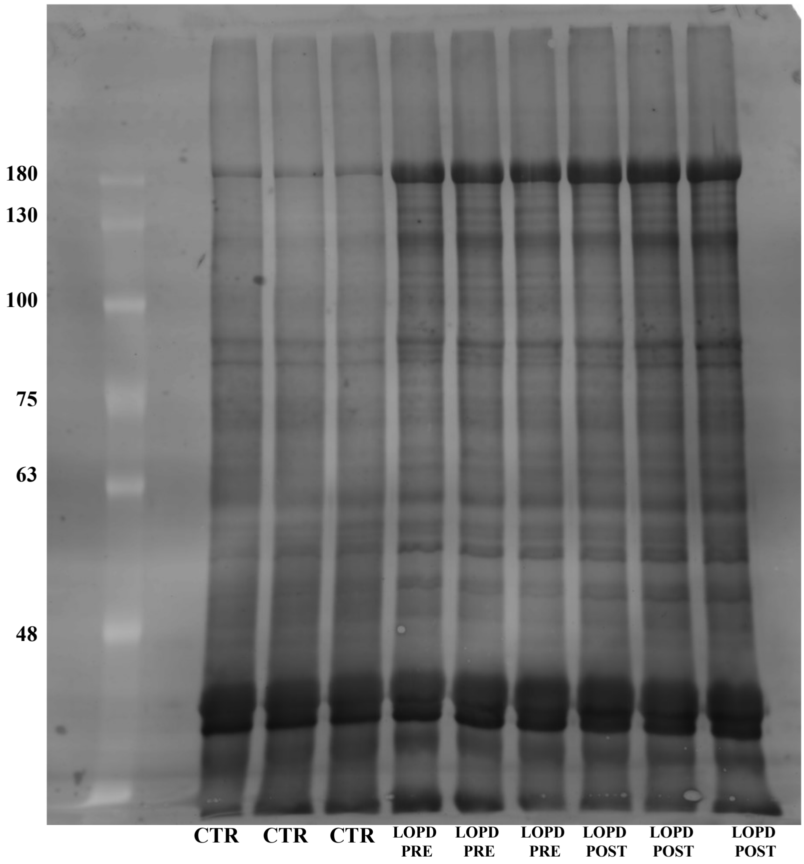

Fig. 7

CHOP (DDIT3 - 27 kDa)
Cell Signaling 2895
gel 10-16%

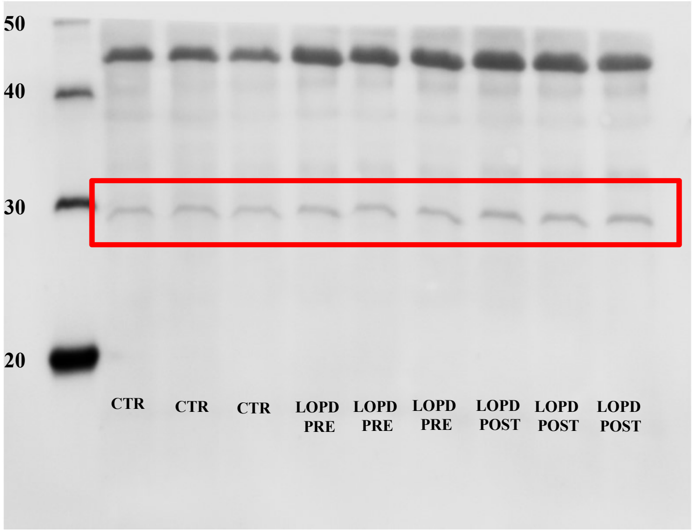

Total stain 10-16%

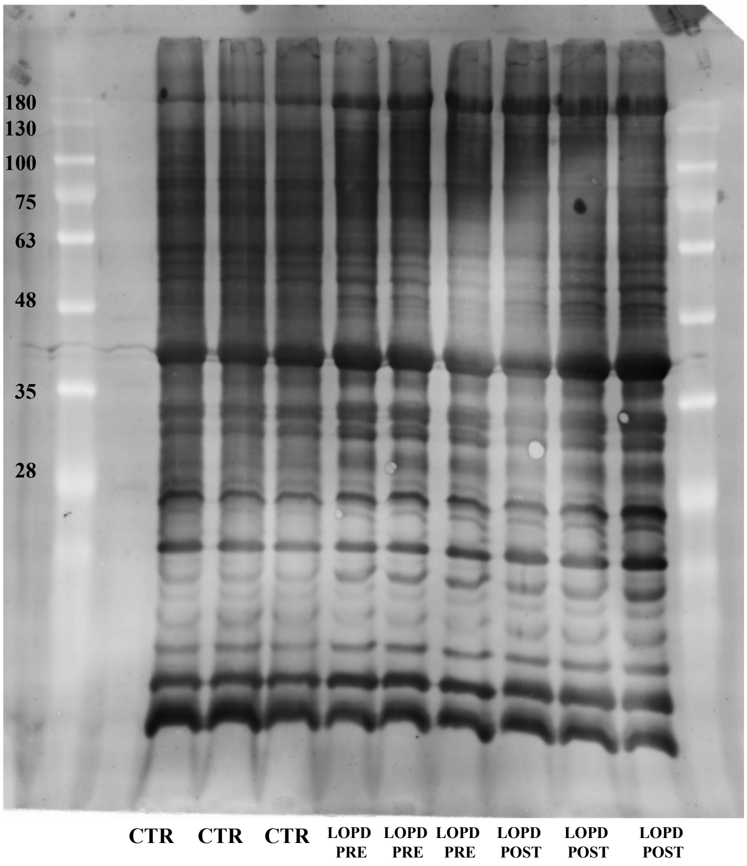

Fig. 8A

LC3B (MAP1LC3B - 14-16 kDa)
Cell Signaling Technology 2775
gel 10-16%

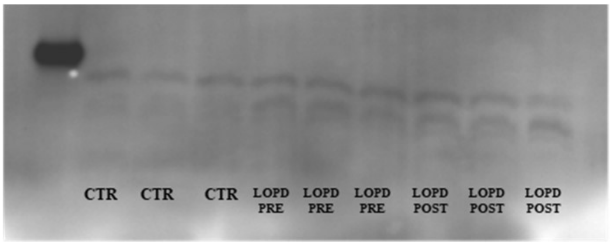

Total stain 10-16%

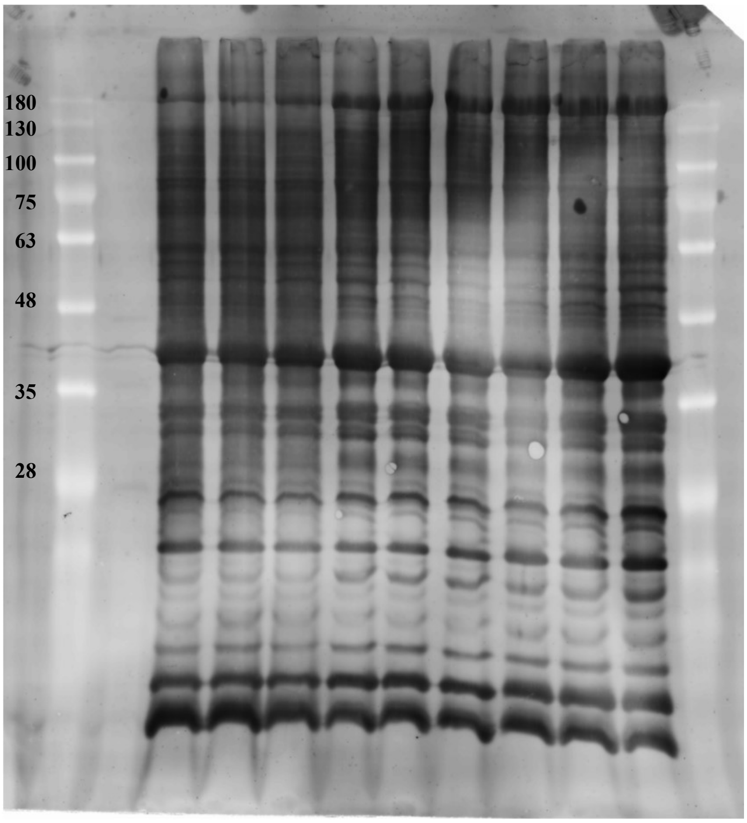

Fig. 8A

SNAP29 (29 kDa)
Santa Cruz Biotechnology sc-135564
gel 12-18%

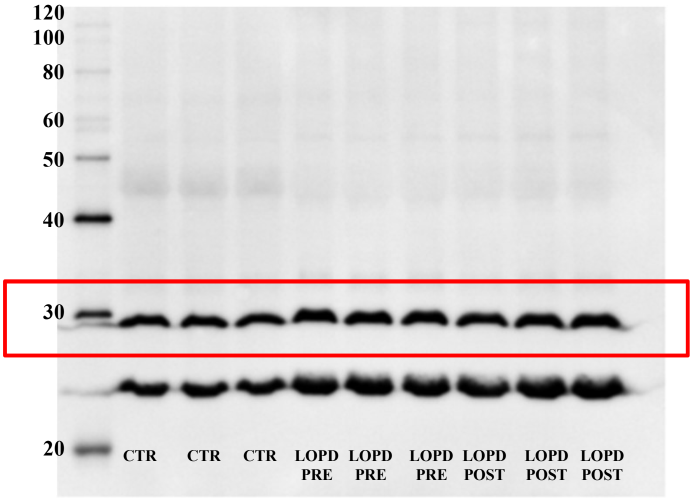

p62 (SQSTM1 - 62 kDa)
Sigma-Aldrich P0067
gel 12-18%

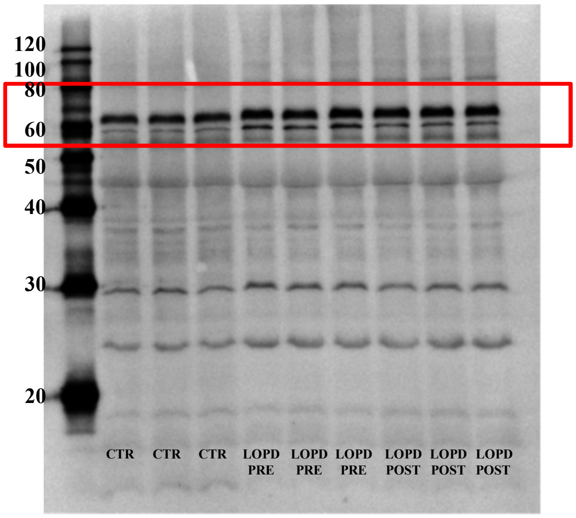

STX17 (33 kDa)
Sigma-Aldrich HPA001204
gel 12-18%

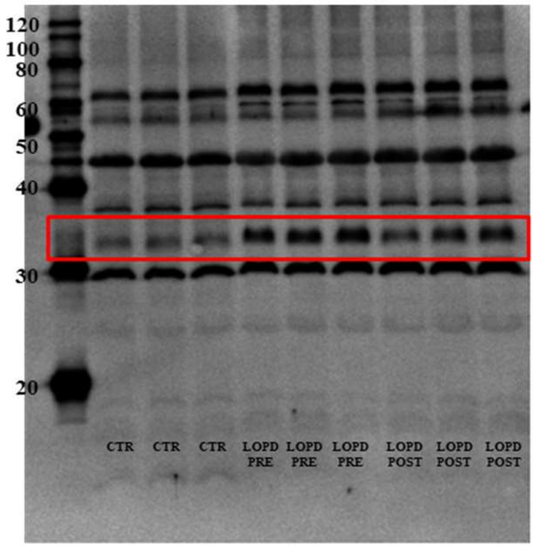

Total stain 12-18%

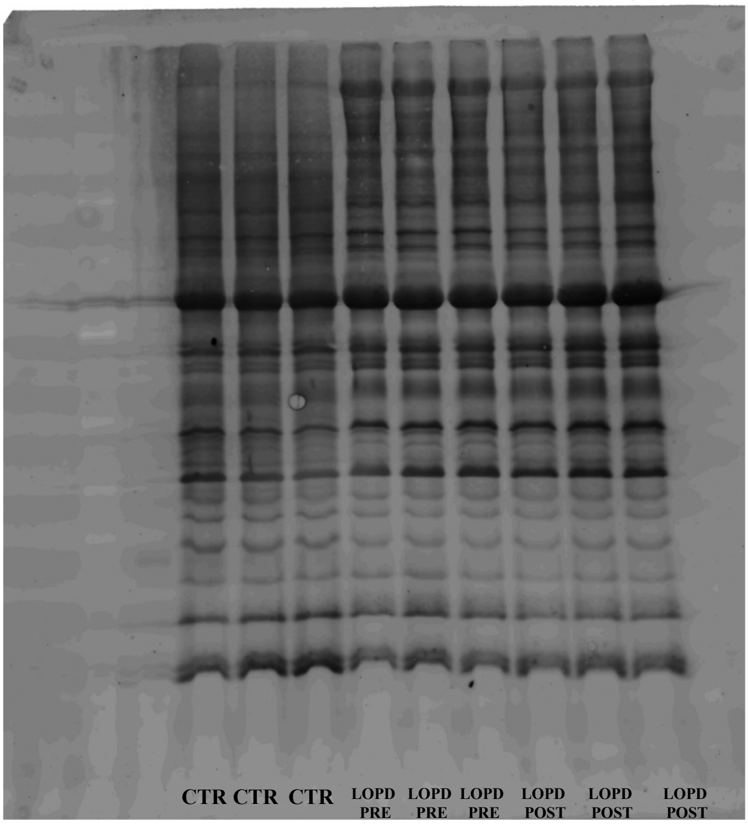

Fig. 8A

VAMP8 (11 kDa)
Santa Cruz Biotechnology sc-166820
gel 12-18%

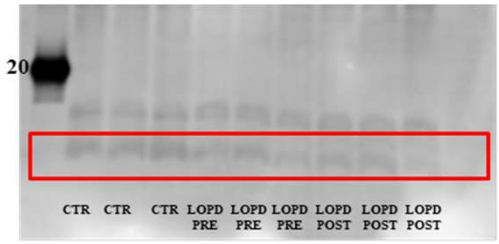

LAMP2 (100-130 kDa)
Cell Signaling Technology 49067
gel 12-18%

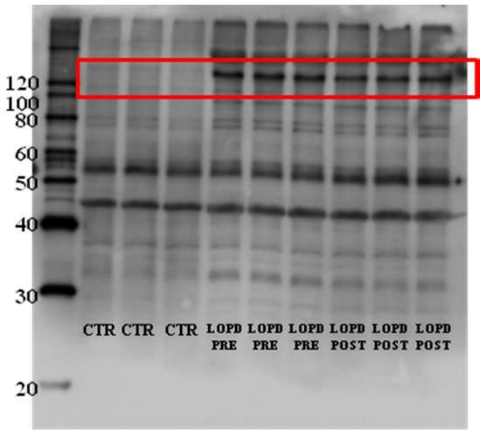

GORASP2 (55 kDa)
Sigma-Aldrich HPA035275
gel 12-18%

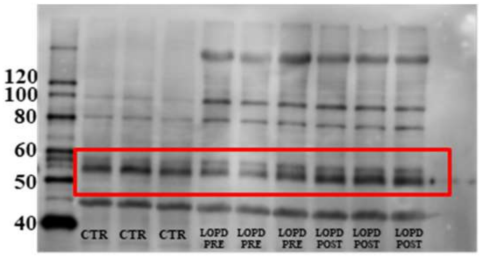

BNIP3 (30-60 kDa)
Sigma-Aldrich B7931
gel 12-18%

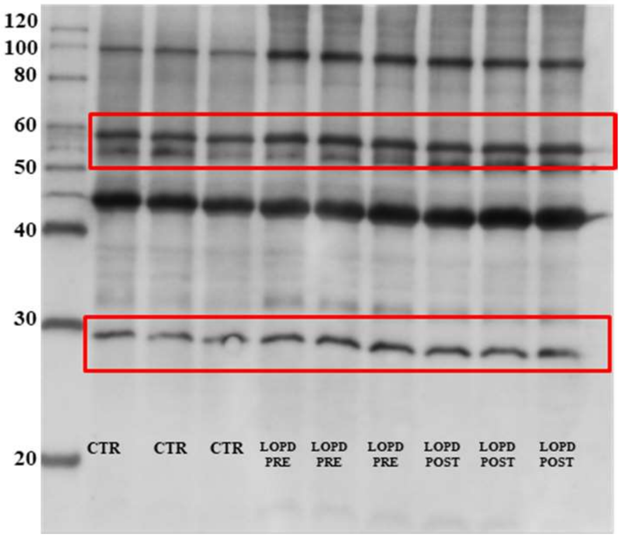

Total stain 12-18%

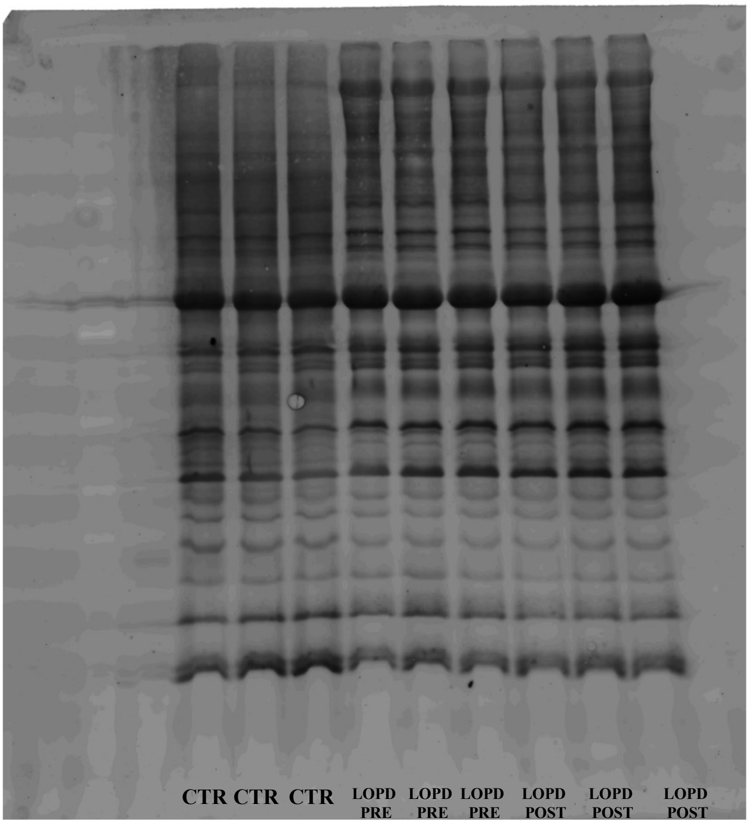

Fig. 8B

Phospho-AMPK (Phospho-PRKAA1 - 62 kDa)
Cell Signaling Technology 2531
gel 8%

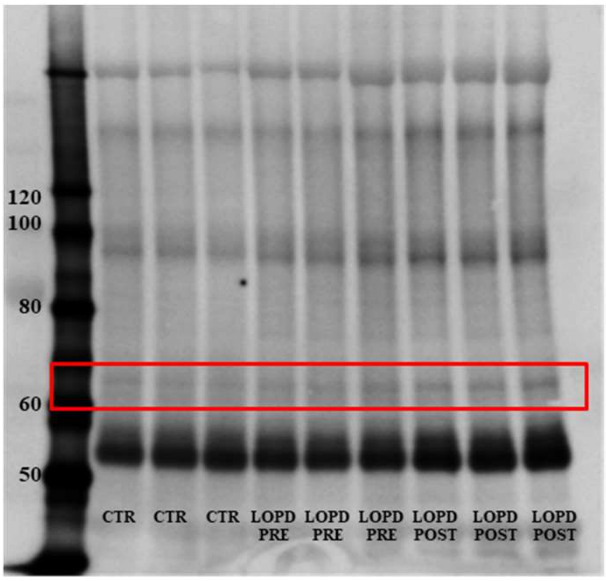

AMPK (PRKAA1 - 62 kDa)
Cell Signaling Technology 2532
gel 8%

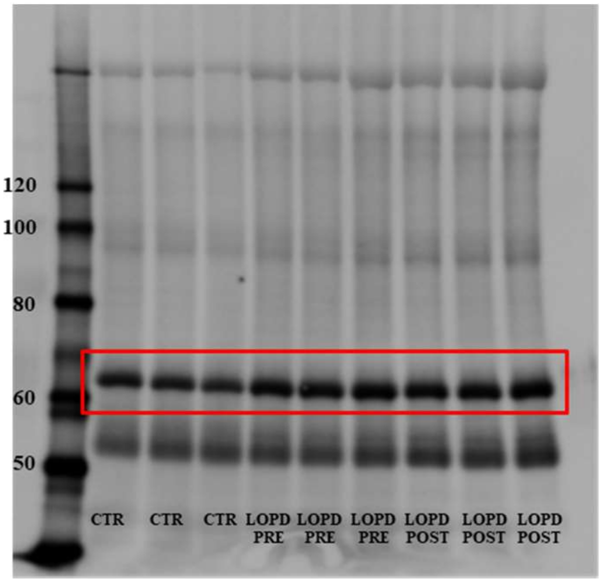

Total stain 8%

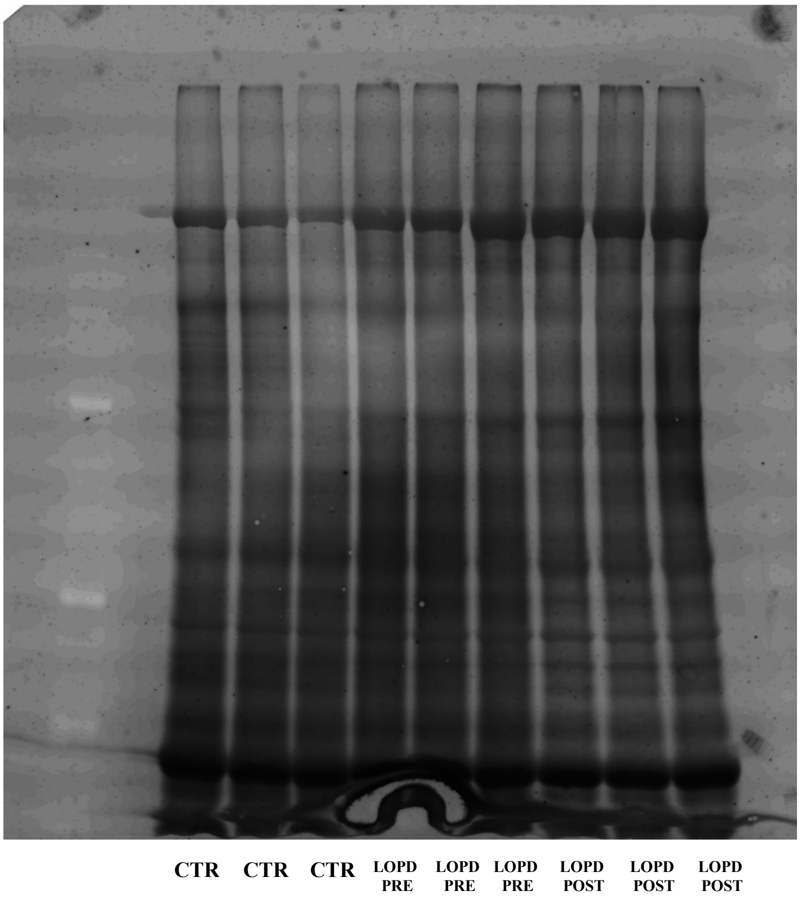

Fig. 8B

Phospho-p3β8 (Phospho-MAPK11 - 43 kDa)
Cell Signaling Technology 9215
gel 12%

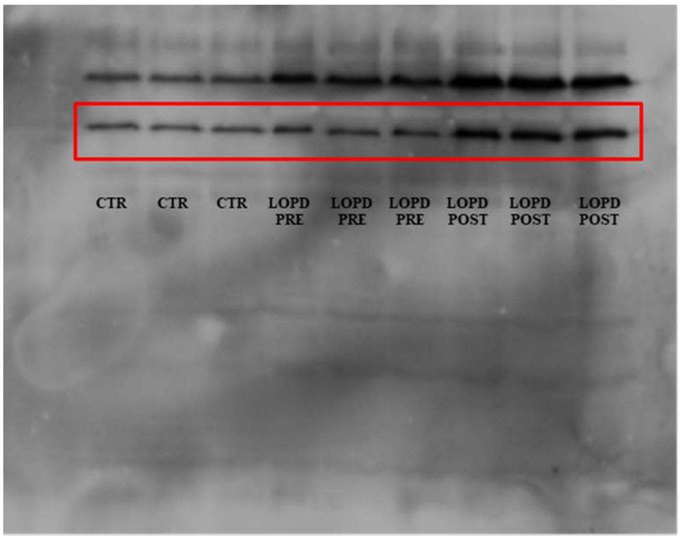

p38β (MAPK11 - 40 kDa)
Cell Signaling Technology 8690
gel 12%

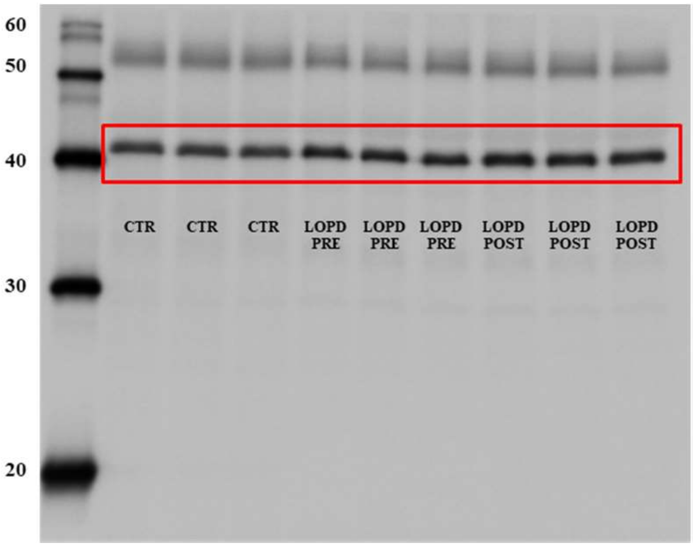

Total stain 12%

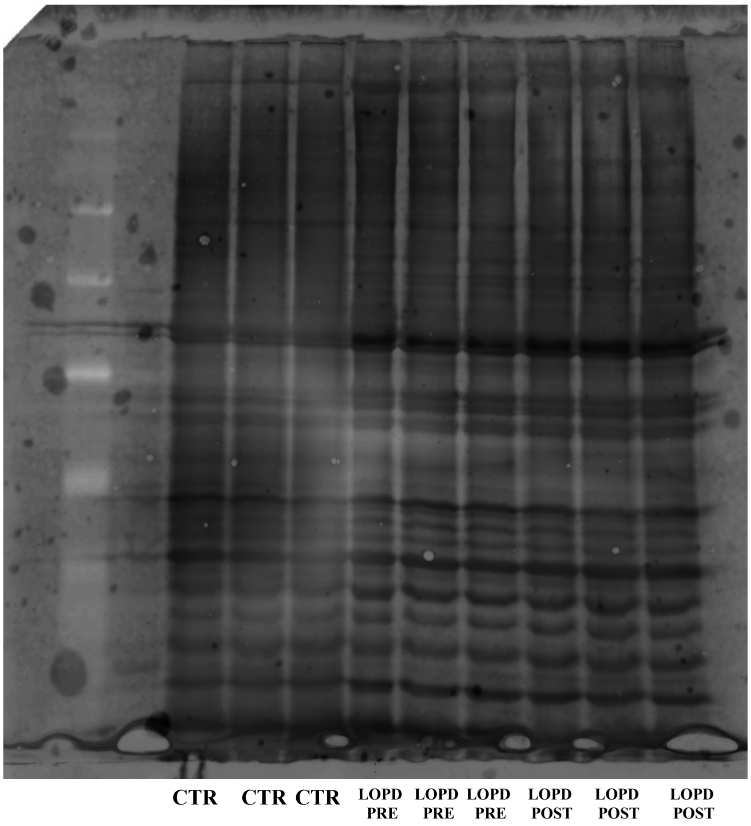

Fig. 8B

Phospho-FoxO3 (95 kDa)
Cell Signaling Technology 9464
gel 12%

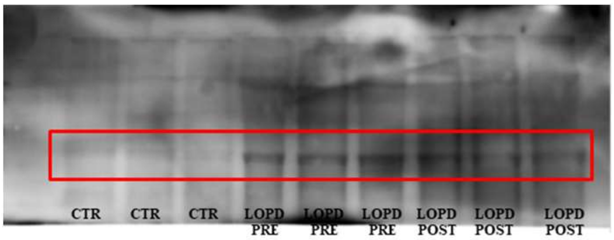

FoxO3 (82-97 kDa)
Cell Signaling Technology 2497
gel 12%

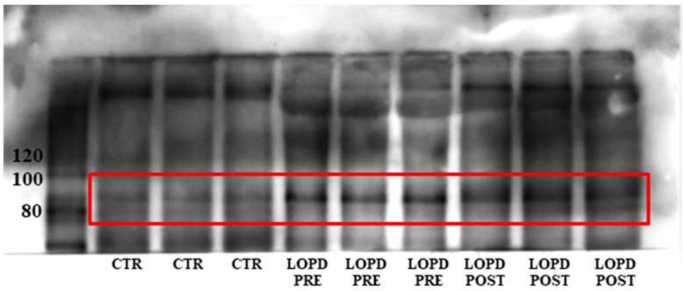

Total stain 12%

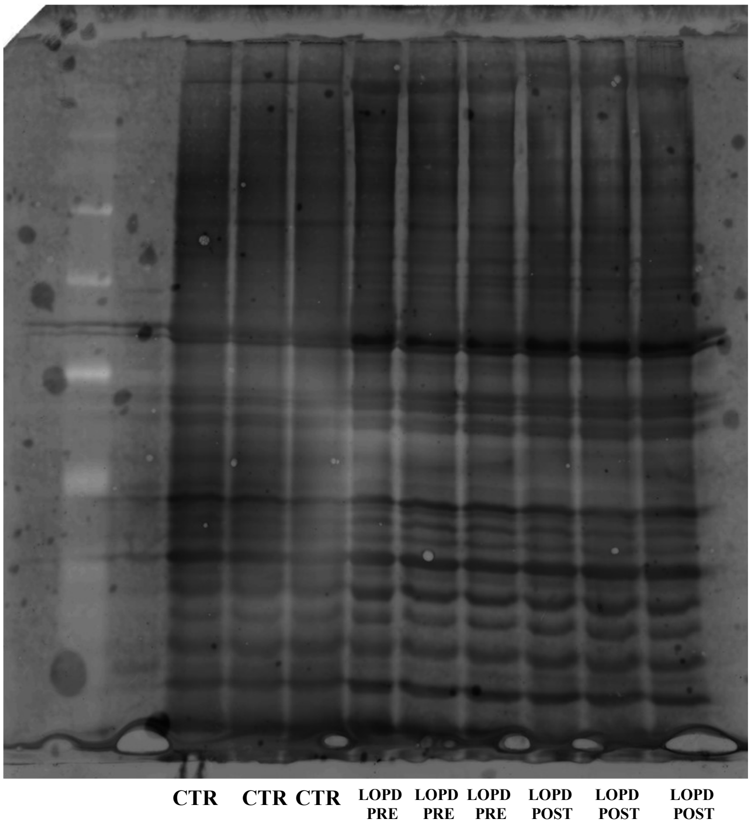

Supplement: Supplementary file 1 [file ijms-22-02850-s001.zip › Supplementary Tables and Figures/Figure S2.pdf]
